# Delayed Diagnosis of a Charcot–Marie–Tooth Disease Type 1–Like Demyelinating Neuropathy in Somalia: A Case Report and Review of Clinical Indicators in Resource‐Limited Settings

**DOI:** 10.1155/crnm/8719531

**Published:** 2026-10-28

**Authors:** Khalid Abdirahman Ahmed, Zekariya Bedru, Salah Abdikarim Abdilahi

**Affiliations:** ^1^ School of Medicine and Surgery Amoud University Borama Somalia amouduniversity.org; ^2^ Department of Internal Medicine Alhayat Hospital Borama Somalia; ^3^ College of Medicine Adal Medical University Borama Somalia medcol.mw

**Keywords:** Charcot–Marie–Tooth disease, consanguinity, nerve conduction studies, peripheral neuropathy, resource-limited setting, Somalia

## Abstract

Charcot–Marie–Tooth (CMT) disease is the most common inherited peripheral neuropathy, but it is rarely reported from the Horn of Africa, and genetic testing is not available in Somalia. We describe a 53‐year‐old Somali man, born to consanguineous parents, with a 43‐year history of slowly progressive distal limb weakness and gait impairment that began at about 10 years of age and had never been medically evaluated; his family attributed his stepping gait to trauma and mountain climbing. His siblings were examined and were unaffected, and his parents were deceased. Examination showed bilateral pes cavus with hammer and clawed toes, marked distal lower‐limb wasting with an inverted champagne‐bottle appearance (more severe on the right), foot drop, distal loss of vibration and proprioception and generalised areflexia, without nerve thickening, tremor, hearing loss or visual loss. Median and ulnar motor conduction velocities were 20‐21 m/s, with prolonged distal and F‐wave latencies, reduced amplitudes and absent lower‐limb motor and sural responses, indicating a demyelinating‐predominant sensorimotor polyneuropathy with secondary axonal loss. Glycated haemoglobin, vitamin B12, thyroid‐stimulating hormone and HIV testing did not identify a common acquired cause. Because genetic testing was unavailable, we diagnosed a probable hereditary demyelinating neuropathy with a CMT Type 1–like phenotype; a recessive form or a de novo variant cannot be excluded. He received ankle–foot orthoses, physiotherapy and counselling, but benefited little, and his function later declined. Childhood‐onset distal wasting with foot deformity, areflexia and uniformly slow conduction velocities should prompt suspicion of CMT even when the family history is negative and genetic testing is unavailable.

## 1. Introduction

Charcot–Marie–Tooth (CMT) disease is a genetically heterogeneous group of inherited peripheral neuropathies characterised by progressive distal muscle weakness and wasting, sensory loss and skeletal deformities such as pes cavus [1]. It affects approximately 1 in 2500 people worldwide [2, 3]. Demyelinating CMT (CMT Type 1) is the most common form in most series, and duplication of the peripheral myelin Protein 22 (*PMP22*) gene, which causes CMT1A, is its most frequent cause [4, 5].

In high‐resource settings, the diagnosis is usually confirmed by molecular genetic testing, increasingly by next‐generation or whole‐genome sequencing [6, 7]. Data from Africa are scarce: A systematic review identified only 107 families with CMT reported across the continent up to April 2021, most of them from North Africa [8], and we did not identify any published case from Somalia. In Somalia, clinicians face substantial barriers to diagnosis, including a lack of specialised neuromuscular services and genetic testing facilities, so many patients experience profound diagnostic delays.

Here, we report an adult Somali man with a clinically and electrophysiologically diagnosed, genetically unconfirmed demyelinating neuropathy with a CMT Type 1–like phenotype, who first came to medical attention more than 4 decades after symptom onset, and we discuss the clinical indicators that can support the diagnosis when genetic testing is unavailable [8, 9].

## 2. Case Presentation

A 53‐year‐old Somali man was first evaluated on 19 February 2026 because of a lifelong, slowly progressive gait disturbance (timeline in Table 1). His difficulty walking began at about 10 years of age with insidious instability and a stepping gait. His family attributed this to trauma, and because he habitually climbed mountains with the help of a walking stick, no one suspected an underlying disease. He had never consulted a clinician about the problem, so there were no earlier diagnoses, investigations or treatments. Over the following 4 decades, he developed progressive thinning of the lower limbs, frequent falls and declining manual dexterity.

**TABLE 1:** Timeline of the case.

Time	Event
About age 10 years	Onset of insidious gait instability and stepping gait; attributed by the family to trauma and mountain climbing; no medical evaluation
Ages 10–53 years	Slowly progressive distal lower‐limb wasting, frequent falls and declining manual dexterity; no clinician consulted
19 February 2026 (age 53)	First medical evaluation: history, examination, laboratory tests, nerve conduction studies and needle EMG; clinical diagnosis of probable hereditary demyelinating neuropathy (CMT1‐like)
After initial evaluation	Bilateral ankle–foot orthoses fitted; physiotherapy started; counselling on neurotoxic drugs and on inheritance
Follow‐up	Little benefit from the orthoses; decline in functional status

His parents were consanguineous. Both had died and had not been examined, and no relative was known to have had similar symptoms. All of his brothers and sisters were examined and had no symptoms or signs of neuropathy, so there was no family history of a similar illness.

Physical examination revealed marked atrophy of the peroneal and gastrocnemius muscles, producing a characteristic stork‐leg (inverted champagne‐bottle) appearance that was more severe in the right leg (Figure 1). Symmetrical skeletal deformities, including bilateral pes cavus with hammer (clawed) toes and foot drop, were prominent (Figure 2). Neurological examination showed 2/5 power in ankle dorsiflexion and 4/5 power in the intrinsic hand muscles. Sensory examination demonstrated a loss of vibration and proprioception in a glove‐and‐stocking distribution. Deep tendon reflexes were globally absent. There was no peripheral nerve thickening, tremor, hearing loss or visual loss.

**FIGURE 1:**
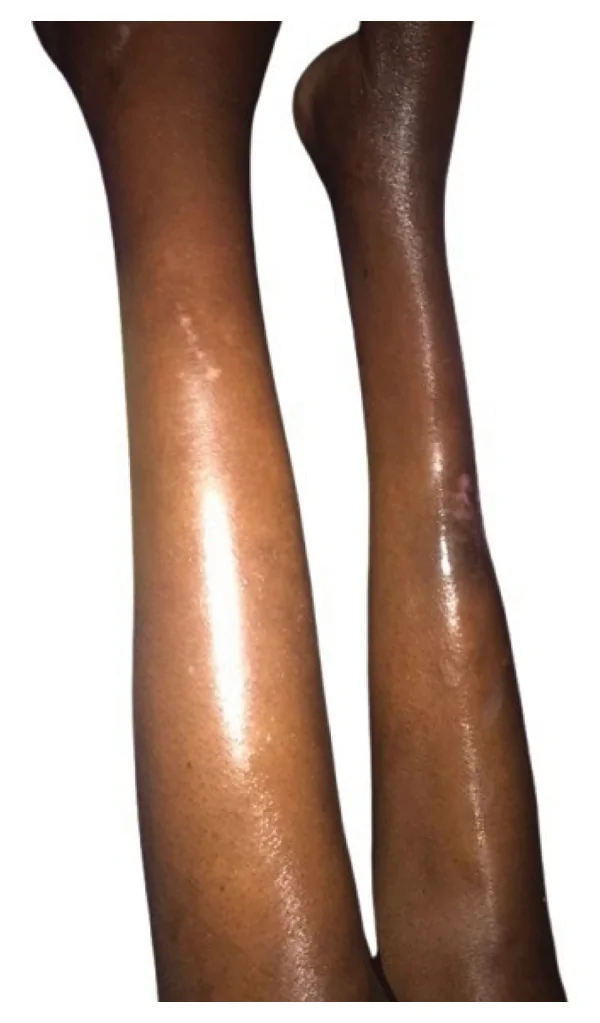
Both lower legs showing marked distal wasting with an inverted champagne‐bottle (stork‐leg) appearance. Wasting was more severe on the right side on examination.

**FIGURE 2:**
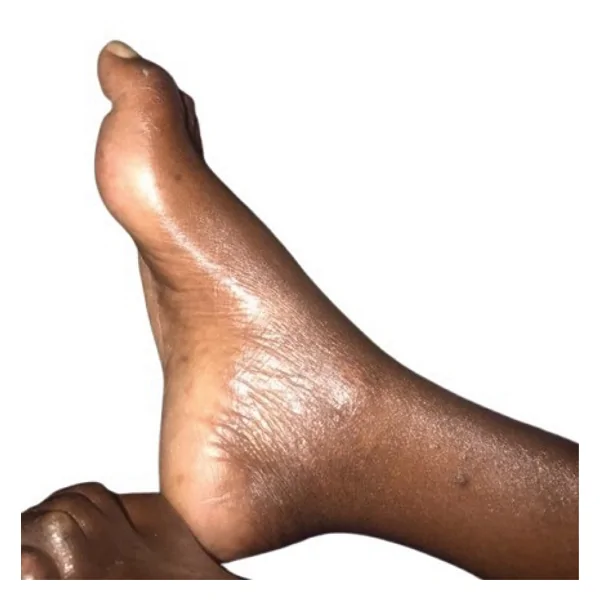
Side view of a foot showing a high longitudinal arch (pes cavus) with toe deformity. Images are deidentified and published with the patient’s written consent.

Basic laboratory tests did not identify a common acquired cause of neuropathy: glycated haemoglobin was 5.8%, serum vitamin B12 was 1176.78 pg/mL, thyroid‐stimulating hormone was 3.1 mIU/L and HIV serology was negative.

Nerve conduction studies (NCS) and needle electromyography (EMG) were performed. The original waveforms were not retained, so the results are given in Tables 2, 3, and 4. Motor conduction velocities were severely and uniformly slowed (median 20.1–20.4 m/s, ulnar 21.3 m/s, with near‐identical values on the two sides), with prolonged distal latencies (7.69–8.60 ms) and F‐wave latencies (53.8–56.2 ms) and reduced compound muscle action potential amplitudes (1.0–1.1 mV). Peroneal and tibial motor responses were absent (Table 2). Sensory conduction velocities were similarly reduced (median 22.5 m/s, ulnar 21.7 m/s) with low amplitudes (2.3–2.6 μV), and the sural response was absent (Table 3). Needle EMG showed high‐amplitude, polyphasic motor unit action potentials with reduced recruitment in the first dorsal interosseous and tibialis anterior, consistent with chronic reinnervation, and no spontaneous activity (Table 4). Together, these findings indicate a severe sensorimotor polyneuropathy that is predominantly demyelinating (velocities far below 38 m/s) with superimposed axonal loss (reduced amplitudes, inexcitable lower‐limb nerves and chronic neurogenic motor unit changes).

**TABLE 2:** Motor nerve conduction studies (NCS).

Nerve (side)	Distal latency (ms)	Amplitude (mV)	Velocity (m/s)	F‐wave (ms)
Median (RT)	8.52	1.1	20.4	55.8
Median (LT)	8.60	1.0	20.1	56.2
Ulnar (RT)	7.69	1.1	21.3	53.8
Peroneal (LT)	—	Absent	Absent	Absent
Tibial (LT)	—	Absent	Absent	Absent

**TABLE 3:** Sensory nerve conduction studies (NCS).

Nerve (side)	Peak latency (ms)	Amplitude (μV)	Velocity (m/s)
Median (LT)	5.8	2.3	22.5
Ulnar (LT)	5.7	2.6	21.7
Sural (RT)	—	Absent	Absent

**TABLE 4:** Needle electromyography (EMG) summary.

Muscle group	Spontaneous activity	MUAP configuration	Recruitment
Biceps brachii	None	Normal	Full
1st dorsal interosseous	None	Polyphasic, high amplitude	Reduced
Tibialis anterior	None	Polyphasic, high amplitude	Absent/reduced

Given the childhood onset, symmetrical distal wasting with skeletal deformities, generalised areflexia and uniformly slow conduction velocities, and the absence of an alternative cause on basic testing, we made a clinical diagnosis of probable hereditary demyelinating neuropathy with a CMT Type 1–like phenotype. Acquired demyelinating neuropathy, such as chronic inflammatory demyelinating polyradiculoneuropathy (CIDP), was considered unlikely because of the lifelong course and the uniform rather than multifocal slowing, and poliomyelitis sequelae were considered unlikely because of the symmetrical sensory loss and generalised areflexia. Genetic testing was not available, so the CMT subtype and the mode of inheritance could not be determined.

The clinical management focused on functional preservation and the prevention of secondary complications. He was fitted with bilateral ankle–foot orthoses (AFOs) to address foot drop and gait instability, and a physiotherapy programme was started, consisting of daily stretching of the Achilles tendons to prevent fixed contractures and low‐impact conditioning to maintain joint mobility. He was counselled to avoid neurotoxic medications known to exacerbate CMT, specifically vincristine, nitrofurantoin and amiodarone [10]. He was also counselled that the condition is probably hereditary, that its mode of inheritance could not be established without genetic testing (consanguinity and unaffected siblings raise the possibility of a recessive form) and that no single risk figure could be given for his relatives; genetic testing and formal genetic counselling were advised whenever they become available. At follow‐up, he derived little benefit from the AFOs, and his functional status had declined.

## 3. Discussion

We describe an adult Somali man, who first came to medical attention more than 4 decades after symptom onset, with a probable hereditary demyelinating neuropathy resembling CMT Type 1. The diagnosis rested on the clinical phenotype and NCS rather than on genetic confirmation. We did not identify any earlier published CMT case from Somalia, and reports from Africa as a whole remain scarce [8].

### 3.1. Diagnostic Reasoning Without Genetic Confirmation

Classically, CMT1 is defined by a median motor conduction velocity below 38 m/s, and CMT2 by velocities above this cutoff [8, 11]. Our patient’s median and ulnar velocities of about 20‐21 m/s, symmetrical and similar across nerves, lie well within the demyelinating range and fit the uniform slowing typical of CMT1A [12, 13]. Amplitudes were also reduced, and lower‐limb responses were absent, so the neuropathy has mixed features, with demyelination predominating. This is expected in long‐standing demyelinating CMT: length‐dependent axonal degeneration accumulates over the years and, more than demyelination itself, correlates with weakness and disability in CMT1A [3, 14]. After more than 40 years of disease, low amplitudes and inexcitable lower‐limb nerves therefore do not argue against a primary demyelinating process, and we regard the axonal loss as secondary. In contrast, an Eritrean man reported from Ethiopia had an axonal‐predominant mixed neuropathy and, after genetic testing, a diagnosis of CMT2A (with copy‐number changes in *MFN2* and *MPZ*) [9]. Both patients had pes cavus, areflexia and no family history, but conduction slowing was the dominant abnormality in ours, which shows how electrophysiology can point towards a CMT category even though it cannot replace genotyping.

Acquired neuropathies were considered. CIDP can mimic CMT and is treatable, so it matters to distinguish it [15]; its electrodiagnostic hallmarks are multifocal features such as conduction block and temporal dispersion, whereas the symmetrical, uniform slowing, lifelong course and pes cavus seen here favour a hereditary process [16]. Poliomyelitis sequelae, a frequent misdiagnosis in this setting, cause asymmetrical flaccid weakness without sensory loss, which does not fit. Basic testing for diabetes, vitamin B12 deficiency, thyroid disease and HIV infection did not identify another cause.

We acknowledge that distinguishing CMT1 from CMT2, intermediate forms and X‐linked or recessive subtypes on clinical grounds alone is difficult, particularly when the findings are mixed. Intermediate conduction velocities (roughly 25–45 m/s), asymmetry and male predominance suggest X‐linked CMT (CMTX1, *GJB1*) [17, 18]; our patient’s velocities were below this range and symmetrical, which argues against, but does not exclude, this possibility. Genetic testing strategies for CMT are guided by conduction velocities and inheritance pattern [12, 19], and this is why we describe the diagnosis as probable and CMT1‐like.

### 3.2. Inheritance Pattern and Family Assessment

Consanguineous parents, unaffected siblings and no family history raise the possibility of an autosomal recessive demyelinating form (CMT4) rather than the autosomal‐dominant *PMP22* duplication that causes most CMT1 worldwide [4, 5]. In the African systematic review, inheritance was autosomal recessive in 91.2% of the 107 families, with *GDAP1*, *SH3TC2* and *LMNA* the most frequently implicated genes, mainly in North African populations [8]. In Türkiye, a country with a high rate of consanguineous marriage [20], CMT4 was the second most common category after CMT1, and recessive disease was more severe, with wheelchair use more frequent than in dominant forms [21]. Recessive axonal forms, such as *SORD*‐related neuropathy, are also increasingly recognised and may be potentially treatable [22]. Alternatively, a de novo–dominant variant, such as a new *PMP22* duplication, could explain a negative family history [1]. These possibilities cannot be separated without genetic testing.

The distinction matters for counselling. A dominant variant carries a 50% risk to each child, whereas in recessive disease, each sibling of an affected person has a 25% chance of being affected, and the children of an affected person are at low risk unless the partner is a carrier, which is more likely in consanguineous communities. Because the pattern was uncertain, we did not give a single transmission risk. Examination of relatives is a feasible first step when genetic testing is unavailable [1, 13]: in our patient, all siblings were examined and were unaffected, which supports a recessive or sporadic origin, although the deceased parents could not be assessed. Where resources allow, NCS of apparently unaffected relatives can reveal subclinical disease and help to separate dominant from recessive or sporadic inheritance.

### 3.3. Diagnostic Delay in Resource‐Limited Settings

The four‐decade delay in our patient had several causes. His symptoms were attributed to trauma and an active mountain lifestyle, no clinician was consulted, and neuromuscular expertise and electrodiagnostic and genetic testing are scarce. The African systematic review similarly linked the paucity of data to the limited availability of neurologists and diagnostic tools [8]. Delays also occur once patients reach a clinic: the Eritrean patient described above was first investigated for a central cause with brain and cervical spine imaging, and genetic testing was delayed by cost and local unavailability [9]. At the same time, molecular diagnosis in specialist centres now relies on next‐generation and whole‐genome sequencing, which increases the diagnostic yield [6, 7], so the gap between well‐resourced and resource‐limited settings is widening.

Pattern recognition can shorten such delays. Four features should prompt suspicion of CMT: a childhood‐onset stepping gait or recurrent falls, symmetrical distal wasting (an inverted champagne‐bottle appearance) with pes cavus or hammer toes, distal sensory loss with generalised areflexia and uniformly slow conduction velocities below 38 m/s. Their presence should lead to referral for NCS and examination of relatives rather than to attribution to trauma or polio.

### 3.4. Management

No disease‐modifying therapy is currently available for any form of CMT, and management is supportive: rehabilitation, orthoses, orthopaedic surgery for deformity and avoidance of neurotoxic drugs [3, 23]. Foot deformities are common in CMT, and orthopaedic assessment should be considered where available [24]. AFOs are the mainstay for foot drop, but their benefit varies; in our patient, the response was minimal, possibly because of severe distal weakness (2/5 in ankle dorsiflexion) and long‐standing deformity. Avoiding drugs such as vincristine, nitrofurantoin and amiodarone remains important [10], particularly where oncology and cardiology drugs are prescribed without knowledge of the patient’s neuropathy.

### 3.5. Strengths and Limitations

The strengths of this report are the detailed phenotype, the clinical photographs, the systematic examination of siblings and the discussion of practical, low‐cost diagnostic steps in a setting with no published experience. Its main limitation is the absence of genetic testing, so the CMT subtype cannot be assigned, and CMT1A, CMT4, CMTX1, intermediate and other hereditary neuropathies cannot be excluded. Parents were deceased, and relatives could not be studied electrophysiologically or genetically. The original NCS waveforms were not retained, only selected nerves were studied, and no nerve biopsy or nerve imaging was available. A validated CMT severity score was not recorded, and the patient’s own perspective on his care was not documented. Longer follow‐up was not available.

In summary, childhood‐onset distal wasting with foot deformity, areflexia and uniformly slow conduction velocities should raise suspicion of a hereditary demyelinating neuropathy in resource‐limited settings, even when there is no family history. Examination of relatives, careful interpretation of NCS and avoidance of neurotoxic drugs are achievable steps, while access to genetic testing remains essential for a definitive diagnosis and accurate counselling.

## Funding

This research received no specific grant or funding from any funding agency in the public, commercial or not‐for‐profit sectors.

## Ethics Statement

Ethical approval was granted by the Amoud University Research Ethics Committee (Reference: AU/REC/2024/MED‐078).

## Consent

Written informed consent was obtained from the patient for the publication of this case report and the accompanying images.

## Conflicts of Interest

The authors declare no conflicts of interest.

## Data Availability

All data supporting the findings are included within the manuscript.
